# Simultaneous Detection of Pathogens and Tumors in Patients With Suspected Infections by Next-Generation Sequencing

**DOI:** 10.3389/fcimb.2022.892087

**Published:** 2022-06-09

**Authors:** Jiachun Su, Xu Han, Xiaogang Xu, Wenchao Ding, Ming Li, Weiqin Wang, Mi Tian, Xiyuan Chen, Binbin Xu, Zhongqing Chen, Jinyi Yuan, Xiaohua Qin, Dongfang Lin, Ruilan Wang, Ye Gong, Liping Pan, Jun Wang, Minggui Wang

**Affiliations:** ^1^ Institute of Antibiotics, Huashan Hospital, Fudan University, Shanghai, China; ^2^ Research and Development Department, MatriDx Biotechnology Co., Ltd, Hangzhou, China; ^3^ The National Clinical Research Center for Aging and Medicine, Huashan Hospital, Shanghai, China; ^4^ Department of Critical Care Medicine, Shanghai General Hospital, Shanghai Jiao Tong University School of Medicine, Shanghai, China; ^5^ Department of Critical Care Medicine, Huashan Hospital, Fudan University, Shanghai, China; ^6^ Department of Critical Care Medicine, Jing’an District Centre Hospital, Fudan University, Shanghai, China; ^7^ Department of Neurosurgery, Putuo District People’s Hospital, Tongji University, Shanghai, China; ^8^ Department of Pathology, Huashan Hospital, Fudan University, Shanghai, China

**Keywords:** pathogen, tumor, infection, copy number variation, next-generation sequencing

## Abstract

**Background:**

Differential diagnosis of patients with suspected infections is particularly difficult, but necessary for prompt diagnosis and rational use of antibiotics. A substantial proportion of these patients have non-infectious diseases that include malignant tumors. This study aimed to explore the clinical value of metagenomic next-generation sequencing (mNGS) for tumor detection in patients with suspected infections.

**Methods:**

A multicenter, prospective case study involving patients diagnosed with suspected infections was conducted in four hospitals in Shanghai, China between July 2019 and January 2020. Based upon mNGS technologies and chromosomal copy number variation (CNV) analysis on abundant human genome, a new procedure named Onco-mNGS was established to simultaneously detect pathogens and malignant tumors in all of the collected samples from patients.

**Results:**

Of 140 patients screened by Onco-mNGS testing, 115 patients were diagnosed with infections; 17 had obvious abnormal CNV signals indicating malignant tumors that were confirmed clinically. The positive percent agreement and negative percent agreement of mNGS testing compared to clinical diagnosis was 53.0% (61/115) and 60% (15/25), *vs.* 20.9% (24/115) and 96.0% (24/25), respectively, for conventional microbiological testing (both *P <*0.01). *Klebsiella pneumoniae* (14.8%, 9/61) was the most common pathogen detected by mNGS, followed by *Escherichia coli* (11.5%, 7/61) and viruses (11.5%, 7/61). The chromosomal abnormalities of the 17 cases included genome-wide variations and local variations of a certain chromosome. Five of 17 patients had a final confirmed with malignant tumors, including three lung adenocarcinomas and two hematological tumors; one patient was highly suspected to have lymphoma; and 11 patients had a prior history of malignant tumor.

**Conclusion:**

This preliminary study demonstrates the feasibility and clinical value of using Onco-mNGS to simultaneously search for potential pathogens and malignant tumors in patients with suspected infections.

## Introduction

It is often difficult to distinguish between malignant tumor exacerbations and infectious diseases based upon clinical manifestations. Symptoms such as fever indicative of infections can occur in patients with malignant tumors. Indeed, the differential diagnosis of a “fever of unknown origin (FUO)” is the most wide-ranging diagnosis in medicine (Fusco et al., 2019). One study, which summarized the frequencies of diagnoses of FUO from selected publications, indicated that infection accounted for 16%-55% of cases, followed by inflammatory (11%-29%), neoplasia (7%-35.4%), undiagnosed illness (7%-51%) and miscellaneous diseases (2.2%-19.8%) (Wright and Auwaerter, 2020). Hematologic tumors, especially lymphoma, are the most common malignant tumors that induce fevers of unknown origin (Zhou et al., 2020). A previous report showed that 58% and 5% of patients with suspected focal infections or inflammation had final diagnoses of infections and malignant tumors, respectively (Bleeker-Rovers et al., 2004). It is well-appreciated throughout medicine that the inability to obtain a timely and accurate diagnosis frequently results in unnecessary antimicrobial treatments, high healthcare costs, and poor prognoses.

Metagenomic next-generation sequencing (mNGS), which has been used in clinical practice for several years, represents an innovative strategy for detecting potential pathogens including bacteria, viruses, fungi, and parasites (Chiu and Miller, 2019; Miller et al., 2019; Wilson et al., 2019). Indeed, studies published to date that explored the clinical applications of mNGS focused primarily on the specific sources of infection, e.g., focal infections, meningitis and pneumonia (Schlaberg et al., 2017; Wilson et al., 2018; Wilson et al., 2019; Zhang et al., 2019). Recent mNGS studies demonstrated that the nucleic acid yields from pathogens account for only 0.00001 to 1–2% of the total reads; human sequences (homo reads) represent >90% (Salzberg et al., 2016; Simner et al., 2018; van Rijn et al., 2019). It is possible to obtain valuable information from this large homo reads database. Chromosome instability, defined as a defect that involves chromosomal gain, loss or rearrangement during tumorigenesis, is a hallmark of cancer (Hanahan and Weinberg, 2011). NGS-based methods used to analyze chromosomal copy number variation (CNV) employ experimental procedures very similar to those used for mNGS.

Considering CNV analysis is already used to assist the diagnosis of genetic diseases and cancer (Zhang et al., 2009; Heitzer et al., 2013), it is conceivable that mNGS homo reads data can be used for tumor detection. This study aimed to explore the clinical value of mNGS for pathogen detection and searching for indications of malignant tumors using homo reads data simultaneously in patients with suspected infections.

## Materials and Methods

### Study Participants

This was a multicenter, prospective case study in which patients were enrolled in four general hospitals in Shanghai, China between July 2019 and January 2020 upon diagnosis of a suspected infection.

Eligible patients fulfilled one of the following criteria: 1) patients with FUO (Petersdorf and Beeson, 1961; Durack and Street, 1991); 2) suspected focal infection (Zhang et al., 2019); 3) infection with standard diagnostic examinations failed to identify an etiological cause within 3 days. Exclusion criteria included difficulty in specimen obtaining and incomplete medical records.

Demographic and clinical data collected for each patient included: sex, age, underlying disease, admission to an intensive care unit (ICU), routine blood examination, clinical microbiological results (cultures for bacteria, acid-fast bacilli and fungi), and pathological reports (pathological types, immunohistochemical markers including CD20, Ki67). Physicians conducted other conventional tests, e.g., tests for EBV and CMV, according to their clinical judgment. Peripheral blood (~5 mL) and clinical fluid samples were collected in Cell-Free DNA Collection Tubes (Cat. CAB0305-100, Lakebio Corp. Hefei, China). All samples were sequenced within two days of collection.

### NGS Sequencing

All samples were subjected to DNA extraction, library preparation, and next-generation sequencing (NGS). DNA extraction and library preparation were conducted on the NGS Automatic Library Preparation System (Cat. MAR002, MatriDx Biotech Corp. Hangzhou, China). Reagents included: Nucleic Acid Extraction Kit (Cat. MD013, MatriDx Biotech Corp. Hangzhou, China), Cell-free DNA Library Preparation Kit (blood samples) (Cat. MD007, MatriDx Biotech Corp. Hangzhou, China) and Total DNA Library Preparation Kit (other samples) (Cat. MD001T, MatriDx Biotech Corp. Hangzhou, China). Libraries were pooled and then sequenced on an Illumina NextSeq500 system using a 75-cycle sequencing kit. A total of 10-20 million reads were obtained for each sample.

### CNV and Pathogen Detection With mNGS Data Simultaneously

Inasmuch as the experimental approach is consistent with current methods of mNGS library preparation, a new analysis procedure (Onco-mNGS) is proposed for pathogen detection and searching for clues of malignant tumors using homo reads data simultaneously (**Figure 2A**).

First, sequencing reads were aligned with the human reference genome (hg19) and only unique, mapped reads were selected for subsequent analysis. The reference genome was segmented into continuous windows of fixed length to calculate the read depth of each window, which was then normalized to the total reads of each sample. Copy number ratios of each window were obtained by dividing the normalized read depth by the average read depth in the reference dataset. Afterwards, the fused lasso method (a generalization of the lasso penalty for sequential signal smoothing with sparsity) was applied to log2-transformed copy number ratios. Smoothed adjacent windows with similar ratios were merged into segments with chromosome positions and average ratios annotated. The copy number of each segment was calculated according to the average ratio and normal copy number of the corresponding chromosomes and then compared with preset thresholds to validate a CNV.

Then, the unmapped reads while aligning against human genome were further used for pathogen detection as followed: firstly, the non-human reads were quickly classified using Kraken2 (Wood et al., 2019) with aligning against the NCBI reference sequence database; Secondly, the classified sequences were aligned against the microbial RefSeq database with bowtie2 (Langmead and Salzberg, 2012) for verification; Next, BLAST (version 2.9.0+) alignment to the nucleotide database was conducted to validate candidate reads, where Kraken2 and Bowtie2 were inconsistent (Zhang et al., 2022). Finally, potential pathogens were selected from the results of previous analysis according to the clinical phenotype; these data were reviewed by senior clinicians.

### Diagnosis of Infection

The clinical diagnosis of infection was determined by the treating team. Two independent senior clinicians in Infectious Diseases analyzed the results of mNGS and conventional microbiological testing and made the adjudication whether the results were relevant to the clinical diagnosis.

### Statistics

Data analyses were performed using SPSS 22.0 software. Baseline characteristics were summarized using descriptive statistics. Categorical variables were compared using the Chi-square test. All *P* values were two-sided, with a *P* value <0.05 considered statistically significant.

## Results

### Patient Characteristics and Pathogen Detection

**Figure 1:**
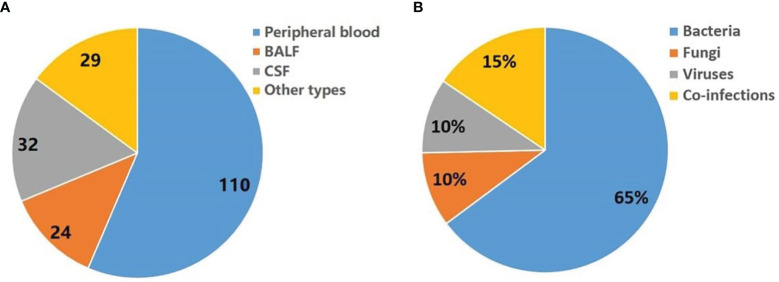
Summary of pathogens detected by mNGS. **(A)** Sample types tested in this study. Other types including sputum, pleural fluid, ascites, and pericardial effusion. **(B)** Distribution of infection types (bacteria, viruses, fungi and co-infection) in 140 patients.

One hundred and forty patients with a 67.9% male to female ratio and a mean age of 55.8 years were enrolled at four hospitals (**Table 1**). Fever or hypothermia was the most frequent symptom observed (77.9%), followed by change in mental status (22.1%), focal pain or dysfunction (18.6%), and cough (17.1%). Most patients (92.8%) came from Intensive Care Units or Departments of Infectious Disease. A total of 92.9% of patients received antimicrobial treatment in the two weeks prior to sample collection. The following samples were collected for mNGS testing from patients recruited for the study: peripheral blood (110 patients), bronchoalveolar lavage fluid (BALF) (24 patients), cerebrospinal fluid (CSF) (32 patients), and other types, e.g., sputum, pleural fluid, ascites, and pericardial effusion (29 patients) (**Figure 1A**).

**Table 1:** Demographic and Clinical Characteristics of the 140 Patients.

Characteristic	Value
Age (years) (mean ± SD)	55.8 ± 20.5
Male sex [n (%)]	95 (67.9)
History of malignancy [n (%)]	33 (23.6)
Immunocompromised status [n (%)]	14 (10.0)
Neutropenia [n (%)]	5 (3.6)
Symptom [n (%)]	
Fever or hypothermia	109 (77.9)
Change in mental status	31 (22.1)
Focal pain or dysfunction	26 (18.6)
Cough	24 (17.1)
Ordering medical team [n (%)]	
ICU	90 (64.3)
Infectious disease	40 (28.6)
Hematology	8 (5.7)
Other	2 (1.4)
Prior antimicrobial agents exposure [n (%)]	130 (92.9)
Anti-bacteria	129 (92.1)
Anti-virus	17 (12.1)
Anti-fungi	26 (18.6)

Seventy-one patients have detected a potential pathogen (environmental microbes which considered as contaminants were filtered), of which bacteria, fungi and virus accounted for 65%, 10% and 10%, respectively. In addition, 15% of patients may have combined infection (**Figure 1B**).

A total of 115 out of 140 patients received a final diagnosis of infection; pneumonia was the most common (66.1%, 76/115), followed by infections of the central nervous system (19.3%, 26/115) and bloodstream (13.9%, 16/115). Twenty-five patients (21.7%) had more than one source of infection. The positive mNGS tests of 61 patients were assessed to be clinically relevant. The positive percent agreement and negative percent agreement of mNGS testing compared to clinical diagnosis was 53.0% (61/115) and 60% (15/25), respectively, *vs.* 20.9% (24/115) and 96.0% (24/25), respectively, for conventional microbiological testing (both *P <*0.01). *Klebsiella pneumoniae* (14.8%, 9/61) was the most common pathogen detected by mNGS, followed by *Escherichia coli* (11.5%, 7/61) and viruses (11.5%, 7/61).

### Analysis of Homo Reads for Tumor Clues

**Figure 2:**
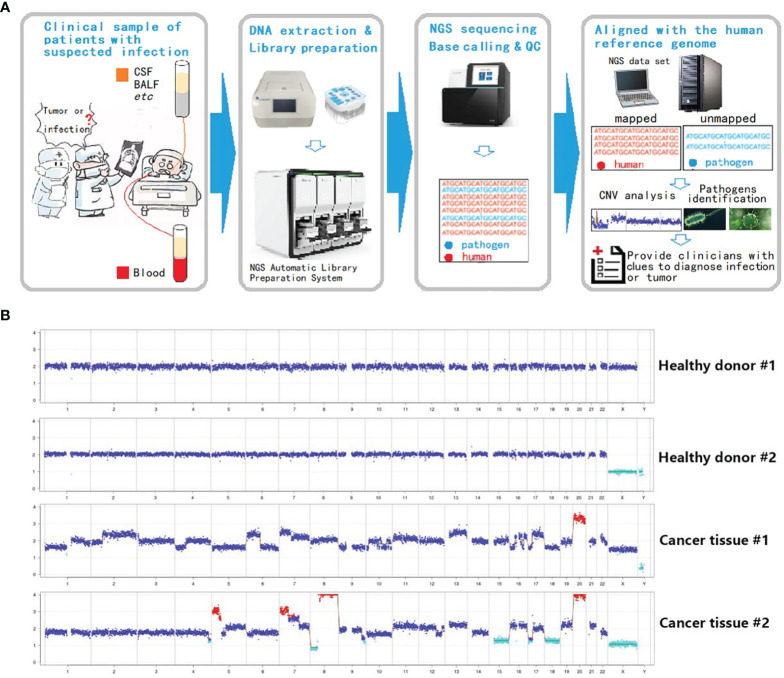
Integration of CNV analysis with mNGS to detect tumors. **(A)** Onco-mNGS procedure. **(B)** CNV data derived from peripheral blood from two healthy donors and two patients with colorectal cancer. Abbreviations: CSF: cerebrospinal fluid; BALF: bronchoalveolar lavage fluid; CNV: copy number variation. Red dots mean the copy number is greater than 2.7; light blue dots mean the copy number is less than 1.3; dark blue dots mean the number of copies is between 1.3-2.7.

This process was tested first using known pathologic samples. Standard diploid samples with no chromosomal deletions or duplications were obtained from healthy individuals (**Figure 2B**). In contrast, dramatic chromosome disorders were observed in tumor tissues obtained from two colorectal cancer patients. Considering the proportion of tumor cells in the samples could be low, the sensitivity of the method was further examined by preparing and testing a variety of samples with different tumor to normal cell ratios (**Supplementary Figure S1**). Chromosome abnormalities were readily detected at ratios of 5-20% supporting the overall concept of using mNGS technologies to identify malignant tumors. These results support the feasibility of integrating CNV analysis with mNGS to detect tumors.

**Figure 3:**
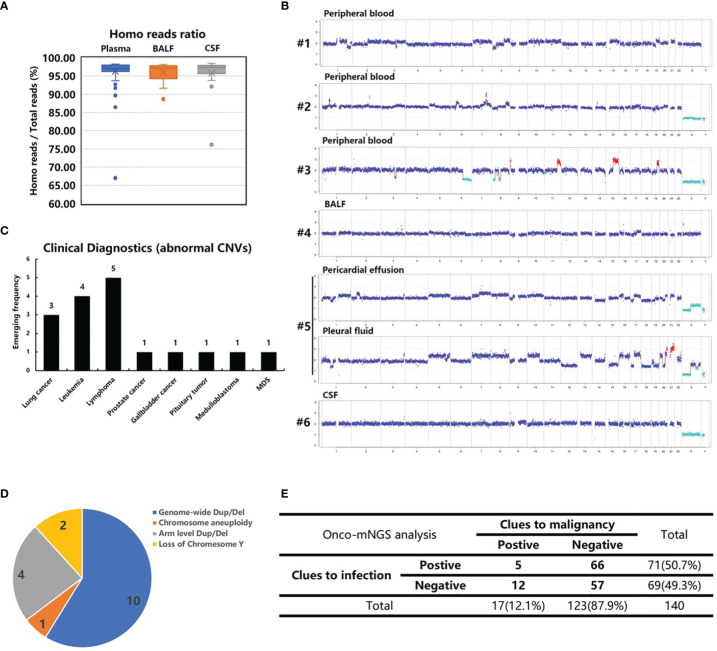
CNV analysis of cancer patients. **(A)** The homo reads ratios of three sample types (peripheral blood, BALF and CSF). **(B)** Abnormal CNVs of patients 1-6 with confirmed malignant tumors who were not recognized before CNV analysis. Patient 6, suspected of having central nerve system lymphoma, refused a biopsy. **(C)** The clinical diagnosis of 17 patients with abnormal CNVs. **(D)** Classification of chromosome abnormalities in 17 patients. **(E)** Summarization of the NGS results of 140 patients with infection and tumor clues.

Next, CNV analysis was performed on each study sample. The homo read ratios were calculated for the three major sample types: peripheral blood, BALF and CSF. The average ratio of homo reads for each sample type was >95% (plasma: 96.26%; BALF: 96.03%; CSF: 95.84%) (**Figure 3A**). After excluding microbial sequences, >8 million homo reads were obtained for each sample, enough to conduct CNV analysis. Seventeen samples with significant chromosomal disorders were identified. Among these, ten were peripheral blood and seven were body fluid samples (three BALF, two pleural fluid, and two CSF). Abnormal CNV signals were observed in both peripheral blood and BALF samples obtained from one patient (#12).

The following chromosome abnormalities were observed in these 17 patients: 1) genome-wide multiple chromosome duplication and deletion in 10 patients (#s 1, 2, 3, 4, 5, 10, 11, 13, 16 and 17); 2) arm level duplication and deletion in four patients (#s 6, 8, 9, 14); 3) loss of chromosome Y in two patients (#s 7 and 12); and 4) chromosome aneuploidy in one patient (# 15) (**Figure 3B** and **Supplementary Figure S2**). The genome-wide chromosome abnormality was observed to be the most type of CNV variation (**Figure 3D**).

### Clinical Validation of Onco-mNGS

Ten peripheral blood samples were obtained from these 17 patients. Additionally, tumor-like signals were found in body fluid samples (three BALF, two pleural fluid, and two CSF). Six patients with obvious, abnormal CNV signals had no recorded history of malignant tumors prior to Onco-mNGS. Malignant tumors were subsequently confirmed in five patients (#s 1-5). Three lung adenocarcinomas and two hematological tumors were determined by pathologic examination of tissues obtained by bone marrow biopsy, bone biopsy or surgery after the results of Onco-mNGS analysis were reported to the attending physicians (**Figure 3C**; **Table 2**). The remaining patient (#6) was suspected of having central nerve system lymphoma, but refused to undergo a biopsy prior to death (**Figure 3**; **Table 2**).

**Table 2:** Clinical characteristics of patients with CNV changes and no history of malignancy before mNGS testing.

No.	Sex, year	History of malignancy	Diagnosis of admission	Diagnosis of discharge	Symptom	Prior antimicrobial agents use	Onco-mNGS		Conventional method	
							Pathogen detected	CNVs	Culture result	Pathology detected
1	Female, 58	None	Thoracic vertebrae lesion	Diffuse large B cell lymphoma	Chest and back pain	Vancomycin; Fosfomycin	None	Multiple Chrs^a^ Del/Dup	None	Bone biopsy: diffuse large B cell lymphoma
2	Male, 67	None	Liver abscess	Lung cancer; Liver occupation; Pneumonia	Fever; Change in metal status	Meropenem; Ciprofloxacin; Linezolid;	None	Chr2 Dup	None	Bronchoscope: neoplasm in superior segmental bronchus Pathology: adenocarcinoma
3	Male, 23	None	Fever of unknown origin	NK T cell lymphoma	Fever; Vomit	Meropenem; Doxycycline; Vancomycin; Cefoperazone; Sulbactam	Blood: *Bacillus thuringiensis*	Multiple Chrs Del/Dup	None	Bone marrow smear: NK T cell lymphoma
4	Female, 71	None	Lung lesion	Lung cancer	Cough; Sputum	None	None	Multiple Chrs Del/Dup	None	Lung biopsy: adenocarcinoma
5	Male, 71	None	Pericardial and pleural effusion	Lung cancer	None	Moxifloxacin	None	Multiple Chrs Del/Dup	None	Lung biopsy: adenocarcinoma
6	Male, 69	None	Encephalitis possible	Central lymphoma possible	Fever; Change in metal status	Ceftriaxone; Metronidazole	CSF: *Elizabethkingia anophelis*	Chr9q Dup	None	–

a Chr, chromosome; Dup, duplication; Del, deletion.

The remaining 11 patients (#s 7-17) with obvious abnormal CNV signals had a history of malignant tumors noted upon chart review (Additional file 1- **Figure S2**; **Table S1**). Hematologic tumors were the most frequently detected accounting for 63.6% of the malignant tumors. In this study, 33 of 140 patients in this study had a history of malignancy before Onco-mNGS analysis; the tumors in 17 of them were evaluated as stable diseases while the remaining 16 patients, including 9 patients with obvious abnormal CNV signals, were suffered from tumor persistence, recurrence or new onset. Overall, the positive percent agreement and negative percent agreement of Onco-mNGS analysis for tumor stage detection was 66.7% (14/21) and 98.3% (116/118), respectively.

By combining the mNGS analyses with CNV analyses, 12 of the 17 patients with obvious abnormal CNV signals were found to have negative mNGS results, indicating malignant tumor may be the cause of infection-like symptoms such as fever (**Figure 3E**).

### Case Vignettes in Which Onco-Mngs Yielded a Malignant Diagnosis

**Figure 4:**
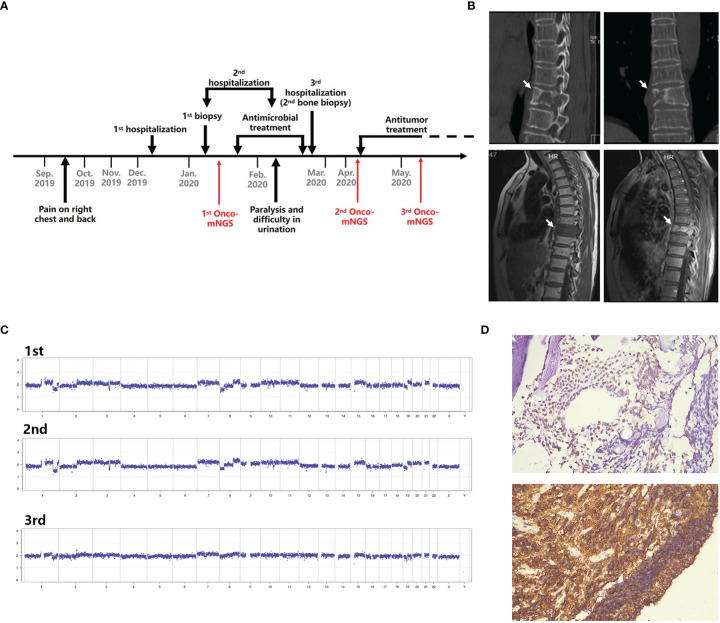
Schematic diagram of Onco-mNGS and a clinical case in which Onco-mNGS was applied and cancer was diagnosed. **(A)** Timeline beginning with the patient’s initial complaint and ending with a final diagnosis. Major events during the course of the patient’s illness are indicated by arrows. **(B)** CT and MRI scans obtained during the patient’s first hospitalization revealed bone destruction of the 9^th^ thoracic vertebrae and right appendix with a paravertebral soft tissue shadow. The lesion is indicated by the white arrow. **(C)** Three CNV results generated at different sampling times. The first and second NGS tests were performed before chemotherapy, and the third one was performed after chemotherapy. **(D)** Results of the repeated bone biopsy showing infiltrating, actively proliferating B lymphocytes evidenced by 60% CD20 and Ki67 positive, consistent with manifestation of diffuse large B cell lymphoma.

Case 1. Diffuse large B cell lymphoma. A 58-year-old female presented after 2 months with right chest and back pain with no fever (**Figure 4A**). A CT scan revealed destruction of the 9^th^ thoracic vertebra and the right appendix with paravertebral soft tissue shadow (**Figure 4B**). Magnetic resonance imaging (MRI) revealed abnormal signals of the 9^th^ thoracic vertebra and right arch with swelling of the surrounding soft tissue. Pathologic results of the bone biopsy indicated infection. The patient was treated empirically with vancomycin and fosfomycin (switched to linezolid due to nausea and vomiting) administered i.v. for 3 weeks. The patient developed paralysis of the lower extremities and difficulty urinating. Repeated MRI showed that the lesion had become significantly larger with spinal compression. Onco-mNGS analysis failed to detect microorganisms in her peripheral blood, but revealed deletions and duplications of large segments in several chromosomes that generated a strong abnormal CNV-based tumor signal (**Figure 4C**). This finding encouraged treating physicians to obtain a second bone biopsy; the result revealed diffuse large B cell lymphoma of the non-germinal center B cell type (**Figure 4D**). The patient received R-CHOP chemotherapy afterwards and the symptoms were mildly relieved. The fluctuation in chromosomal variation was much smoother in the fourth, compared to previous, Onco-mNGS tests suggesting that the proportion of tumor cells had decreased.

Case 2. NK/T cell lymphoma. A 23-year-old male presented after 2 weeks of fever and vomiting. Upon admission, the patient exhibited a low platelet count (45×10^9^/L) and elevated levels of inflammatory markers (C-protein: >199 mg/L; procalcitonin: 13.92 ng/mL). Tests for pathogens that included Epstein-Barr virus (EBV), cytomegalovirus (CMV), cryptococcus, tuberculosis, and parasites were negative; blood cultures for bacteria, acid-fast bacilli, and fungi were also negative. A PET-CT scan showed an enlarged spleen with increased uptake of FDG indicator. The patient underwent two diagnostic bone marrow punctures and a splenectomy; the results, however, did not support a definitive diagnosis. The patient was treated empirically with meropenem, vancomycin, doxycycline, gentamycin, and trimethoprim-sulfamethoxazole administered sequentially, but became worse with progressive hepatic deterioration (total bilirubin: 356.4 µmol/L). The results of a third bone marrow biopsy revealed NK/T cell lymphoma. Onco-mNGS results showed disruptions in several chromosomes indicative of a tumor. This was confirmed by the results of a third bone marrow smear. Unfortunately, this information was not considered *a priori* by the attending physicians and the patient soon died of tumor progression.

Case 3. Lung adenocarcinoma. A 71-year-old female presented after 2 months of cough and sputum, but no fever. Her chest CT scan showed pneumonia; *Mycoplasma pneumoniae*-specific IgM antibody was positive. She was treated with cefuroxime and azithromycin administer i.v. for 6 days, then switched to oral moxifloxacin for 10 days. The patient did not improve in clinical or radiological presentation. Repeated, *M. pneumoniae* serum antibody was negative. Bronchoscopy was conducted, but tests for pathogens including bacteria, acid-fast bacteria, and fungi in BALF were negative. Tumor markers in this patient, i.e., CA199, CEA, CA125, CY211 and SCC, were in the normal range. The patient received a pulmonary lobectomy and the pathological result indicated lung adenocarcinoma.

## Discussion

Suspected infections in patients may have alternate causes that include malignant tumors and autoimmune diseases, namely FUO. Timely identification of the specific cause is critical in treating these patients appropriately. Although improved serologic, laboratory, and imaging technologies has been applied to clinical practice, some prolonged fevers continue to elude a diagnosis often, suggesting that fevers may have too many origins to make significant headway solely through a traditional applied testing algorithm (Wright et al., 2022). mNGS was recently developed for infectious disease diagnostics and can theoretically detect all pathogens in clinical samples. Compared to traditional culture method, mNGS is noted for its enhanced capacity to detect fungi, viruses, anaerobes, and atypical pathogens (a unique ability among clinical approaches to diagnosing infections) (Blauwkamp et al., 2019; Miller et al., 2019; Wilson et al., 2019). Moreover, prior antibiotic treatment is less likely to affect the results of mNGS analysis (Miao et al., 2018). While mNGS exhibits distinct advantages in diagnosing infectious diseases, its use in diagnosing non-infectious diseases has yet to be examined although the vast majority of data derives from the human genome. The current study demonstrates these data represent an extremely valuable diagnostic resource.

Previous studies reported abnormal CNVs suggestive of high genomic instability in cell-free DNA preparations obtained from patients with malignant tumors such as non-small cell lung and prostate cancers (Zhang et al., 2009; Heitzer et al., 2013). Genetic disease CNV and tumor-like CNV are very different. Genetic CNV usually entails duplication or deletion of large chromosomal segments; patients with large deletions and duplications or aneuploidies show serious genetic disease phenotypes at a very early age. CNVs caused by tumors appear as duplications or deletions of large fragments or entire chromosomes; copy number is generally not an integer. The types of anomalies differed among the 17 samples that exhibited significant chromosomal disorders in the current study. Some chromosomes showed global chaos while others showed local fluctuation. This may relate to tumor type and the ratio of tumor cells. Interestingly, loss of the Y chromosome was observed in two cases, but these cases did not show any abnormalities related to the male sex chromosome. Rather, both patients had leukemia. Previous studies report that the Y chromosome is lost 15 to 80% of the time in cases involving a variety of cancer types (Hunter et al., 1993; Park et al., 2006; Bianchi, 2009; Duijf et al., 2013). It is anticipated that the utility of the homo read approach for exploiting mNGS data to support medical diagnoses will continue to increase. This increase will depend upon constantly up-dating the Onco-mNGS procedure to include new insights into CNVs (general, as well as disease-specific, manifestations) gathered from the medical literature.

Diagnosis of malignant tumors often requires invasive measures such as puncture or surgery. Patients often delay examination due to concerns about the necessity and potential harm of invasive procedures. Although the type of information obtained from homo read data using Onco-mNGS methodology does not provide a direct diagnosis, it provides potential diagnostic clues that can persuade patients to receive invasive operations based on risk and benefit considerations, as showed in Case 1 and Case 3. Importantly, combining CNV and mNGS analyses does not increase costs or require additional sampling. With the reduction of sequencing costs and the development of technology, the current cost of mNGS has dropped to an acceptable level, and more and more clinical application scenarios have been demonstrated. In this study, with the help of the automated PCR-free library building platform (NGSmaster, MatriDx Biotech Corp. Hangzhou, China) (Luan et al., 2021), the turn-around time (TAT) can be shorten within 24 hours. Thus, utilization of Onco-mNGS alongside ongoing mNGS testing in hospitals should provide doctors a powerful new tool for making informed and quicker decisions during the diagnosis process.

In this study, 33 of 140 patients had a history of malignancy; only 11 of these patients expressed obvious, abnormal CNV signals, and 9 of which were reported to be suffering from tumor persistence, recurrence or new onset. Considering the relatively short half-life of circulating free DNA (ranging from 16 minutes to 2.5 hours in peripheral blood) (Diehl et al., 2008), the detection of chromosomal abnormalities in these patients suggest an active state of malignancy. It is reasonable to assume, therefore, that tumor cells exist and cause the clinical symptoms, e.g., fever, observed in these 11 patients. The accumulated clues that occur by considering both infection and malignant tumors as causative agents of disease will help formulate more effective treatment strategies. Previous studies also indicated that tumor CNV burden might foretell outcomes such as recurrence and survival (Hieronymus et al., 2018). It will be interesting to investigate Onco-mNGS as an approach to determine residual tumor or relapse in this patient group. In addition, infections are common complications occurred in cancer patients, i.e., cancers are underlying diseases of infections. Onco-mNGS will be helpful to avoid delay of cancer diagnosis for patients with cancer and infection, who is usually diagnosed as infections by traditional culture method or mNGS.

The present study has several limitations. These include the relatively small number of patients with suspected infections that yielded the mNGS data examined herein, and the small number of malignant cases detected. Additionally, the real time disconnect between examination of the mNGS data and the treatment provided by attending physicians is far less than optimal. It is anticipated that the coordinated application of Onco-mNGS data and standard mNGS data analyses going forward will substantially shorten diagnostic time, and thereby increase the utility of this information.

## Conclusions

This study reported a new procedure named Onco-mNGS, based upon mNGS technologies and chromosomal CNV analysis, for simultaneously detection of pathogens and malignant tumors in patients with suspected infections. Onco-mNGS exhibited a significantly higher sensitivity rate for diagnosis of a clinically relevant infection than did traditional microbiological testing, and also enhanced the ability for early differentiation diagnosis of malignant tumor. Further efforts are needed to evaluate the clinical value of Onco-mNGS.

## Data Availability Statement

The datasets (excluding human genomes) presented in this study can be found in online repositories. The names of the repository/repositories and accession number(s) can be found at: https://www.ncbi.nlm.nih.gov/bioproject/PRJNA813350.

## Ethics Statement

The studies involving human participants were reviewed and approved by Ethics Committee of Huashan Hospital (EC Approval No.: 2018-461). The patients/participants provided their written informed consent to participate in this study.

## Author Contributions

JS and XX collected and analyzed clinical data, and wrote the manuscript. XH conducted experiments, analyzed the data, and wrote the manuscript. WD conducted experiments and analyzed the data. ML, WW, MT, XC, BX, JY, XQ, DL, RW, YG, and LP included patients and collected clinical data. ZC had pathological examination. XX, JW, and MW designed the study and revised the manuscript. All authors contributed to the article and approved the submitted version.

## Funding

This work was supported by the National Natural Science Foundation of China (grant numbers 81991531, 81773785, and 81903673), Shanghai Municipal Science and Technology Commission (17411950704, 18411950600), and Shanghai Sailing Program (19YF1405400).

## Conflict of Interest

Author XH, WD, and JW are employed by MatriDx Biotechnology Co., Ltd.

The remaining authors declare that the research was conducted in the absence of any commercial or financial relationships that could be construed as a potential conflict of interest.

## Publisher’s Note

All claims expressed in this article are solely those of the authors and do not necessarily represent those of their affiliated organizations, or those of the publisher, the editors and the reviewers. Any product that may be evaluated in this article, or claim that may be made by its manufacturer, is not guaranteed or endorsed by the publisher.
