# Supplementary material for: Simultaneous Detection of Pathogens and Tumors in Patients With Suspected Infections by Next-Generation Sequencing
Source: Front Cell Infect Microbiol. 2022 Jun 9;12:892087. doi: 10.3389/fcimb.2022.892087 (PMC9218804; doi:10.3389/fcimb.2022.892087)
Supplement: Supplementary file 1 [file DataSheet_1.docx]

**Supplementary Information**


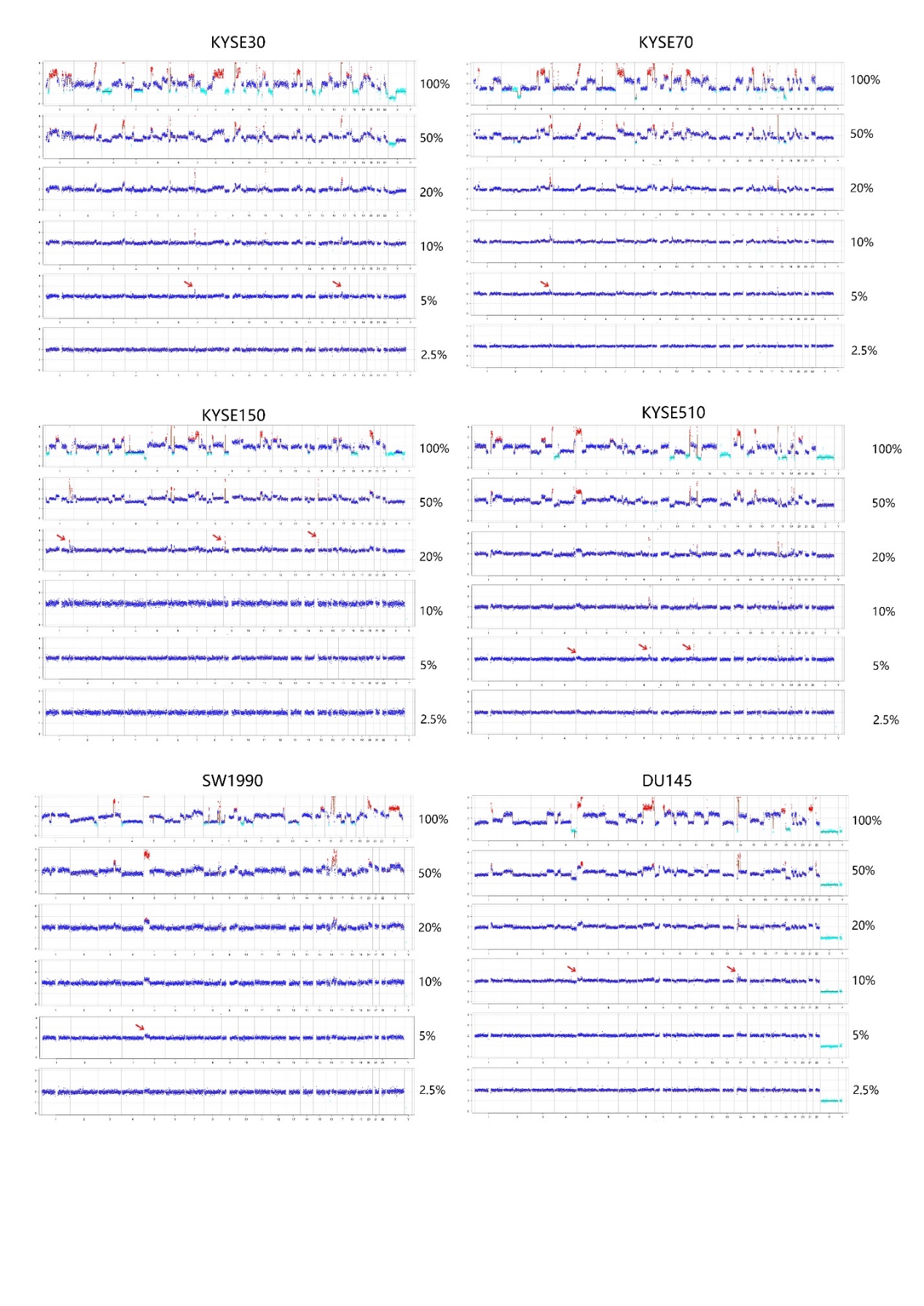


**FIGURE S1.** The sensitivity of Onco-mNGS was tested by mixing six cancer cell lines with normal cells at different ratios. The red arrows show abnormalities detected in samples at the minimum proportion of cancer to normal cell ratio.


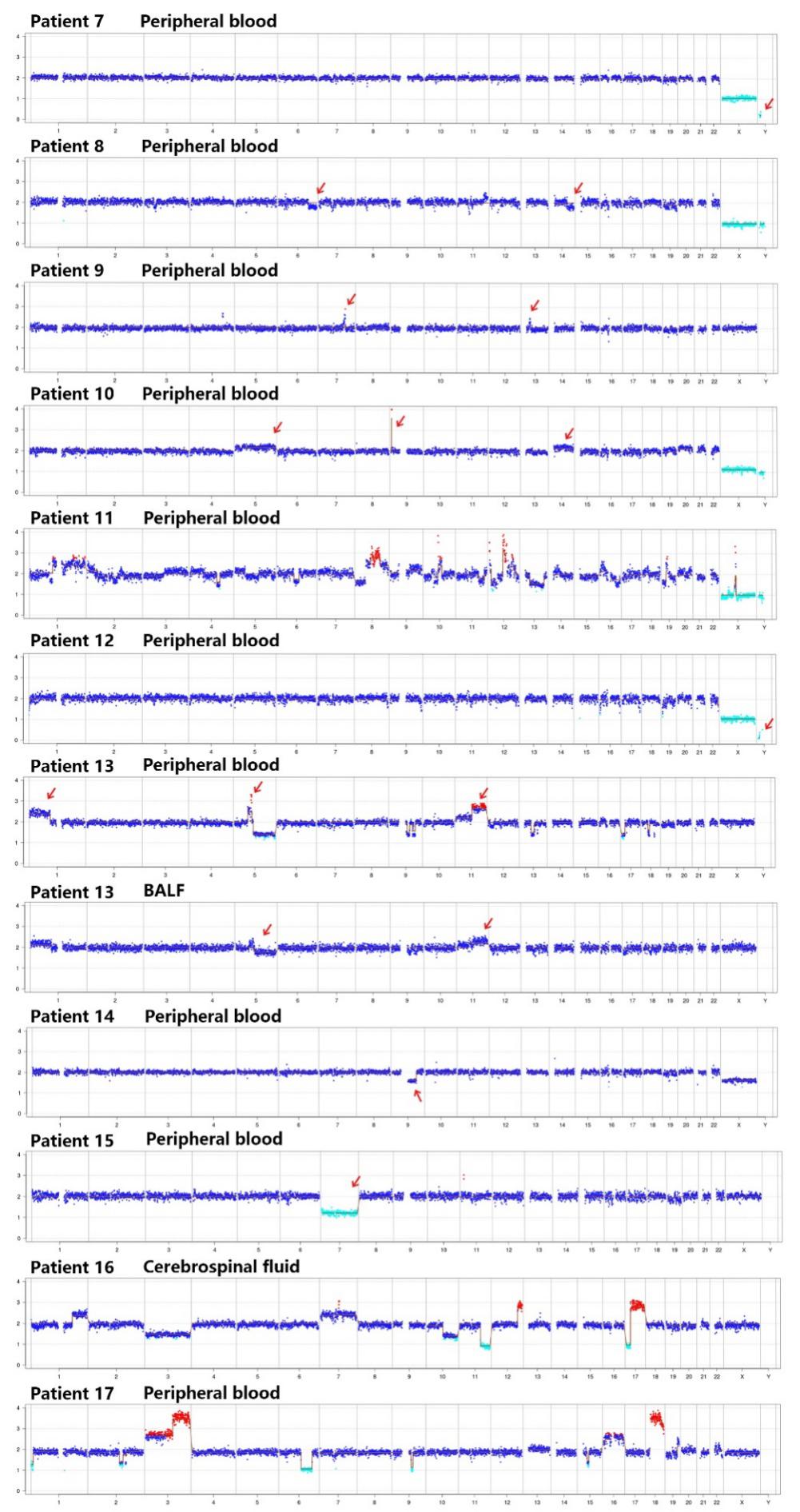


**FIGURE S2.** CNVs of cancer patients. These images show abnormal CNVs of patients (#s 7-17) with histories of malignancy. The red arrows indicate abnormal CNVs.

| **TABLE S1.** Clinical characteristics of the rest 11 patients with CNV changes and a history of malignance | | | | | | | | | |
| --- | --- | --- | --- | --- | --- | --- | --- | --- | --- |
| **No.** | **Sex, year** | **History of malignance** | **Diagnosis before NGS** | **Diagnosis of Discharge** | **Syndrome** | **Prior antimicrobial agents use** | **Clinically relevant pathogen detected in mNGS** | **Clinically relevant pathogen detected in culture result** | **CNVs details** |
| 7 | Male, 62 | Acute promyelocytic leukemia | Pneumonia;  Central nervous system infection | Pneumonia;  Central nervous system infection | Fever;  Change in metal status | Meropenem;  Tigercycline;  Linezonaid; | None | None | ChrY Loss |
| 8 | Male, 59 | Stomach;  Lymphoma | Pneumonia | Pneumonia | None | Ceftriaxone;  Moxifloxacin;  Piperacillin/Tazobactam;  Levofloxacin;  Sulfamonomethoxine/Trimethoprim | Balf: Pneumocystis *jirovecii*  Blood: Pneumocystis *jirovecii* | None | Chr6q Del;  Chr14q Del;  Chr11q Dup |
| 9 | Female, 56 | Gallbladder cancer | Pneumonia | Lung lesion: pneumonia possible, tumor possible | Cough; Sputum;  Weakness | Imipenem;  Doxycycline | Blood: None  Balf: Human parainfluenza virus type 4; Streptococcus *pneumoniae* | None | Chr4q(dup2.4Mb);  Chr7q(dup 4.2Mb) |
| **Patient No.** | **Sex, year** | **History of malignance** | **Diagnosis before NGS** | **Diagnosis of Discharge** | **Syndrome** | **Prior antimicrobial agents use** | **Clinical relevant pathogen detected in mNGS** | **Clinical relevant pathogen detected in culture result** | **CNVs details** |
| 10 | Male, 71 | Lymphoma | Skin and soft tissue infection | T-cell lymphoma possible;  Skin and soft tissue infection ;  Pneumonia | Fever;  Sweat | Piperacillin/Tazobactam;  Cefoperazone/sulbactam;  Levofloxacin; | None | None | Chr5 Dup;  Chr14 Dup;  Chr9p (dup_2.1Mb) |
| 11 | Male, 91 | Prostate cancer with bone metastasis | Pro-operative brain injury | Pro-operative brain injury;  Pneumonia | Fever | Piperacillin/Tazobactam;  Tigercycline | None | None | Multiple Chrs Del/Dup |
| 12 | Male, 59 | None | Acute myeloid leukemia | Acute myeloid leukemia | Fever | Levofloxacin;  Meropenem;  Imipenem;  Posacozole | None | None | ChrY Loss |
| 13 | Female, 56 | Myelodysplastic syndrome | Myelodysplastic syndrome | Myelodysplastic syndrome;  Pneumonia | Fever;  Cough;  Headache | Isoniazid;  Imipenem | BALF: Neisseria *flavescens*;  Blood: None | None | Multiple Chrs del/dup; |
| **Patient No.** | **Sex, year** | **History of malignance** | **Diagnosis before NGS** | **Diagnosis of Discharge** | **Syndrome** | **Prior antimicrobial agents use** | **Clinically relevant pathogen detected in mNGS** | **Clinically relevant pathogen detected in culture result** | **CNVs details** |
| 14 | Female, 55 | None | Acute myeloid leukemia | Acute myeloid leukemia; Bloodstream Infection | Fever;  Cough;  Sputum | Imipenem;  Posacozole;  Vancomycin;  Cefoperazone/sulbactam;  Tigercycline | Blood: Stenotrophomonas *maltophilia* | Blood: Stenotrophomonas maltophilia | Chr9q Dup;  ChrX Del; |
| 15 | Female, 49 | None | Acute myeloid leukemia | Acute myeloid leukemia | Fever | Imipenem;  Posacozole;  Cefoperazone/sulbactam;  Piperacillin/Tazobactam;  Entecavir | None | None | Chr7 Del |
| 16 | Female, 6 | Medullo-  blastoma | Medullo-  blastoma | Central nervous system infection | Fever;  Facial paralysis | Fluconazole;  Fosfomycin; | None | None | Multiple Chrs Del/Dup |
| 17 | Female, 68 | [Pituitary tumor](http://www.baidu.com/link?url=Tpt1JA0TUlDTNzqNUw1heZk_3aYRpOUOfJ0BjCohLiGpJw8b10iDhPESgfPqToE4VNJmChT5KaXWZzVx9mV4W6_dTaVrh-dH1-1qkWDfLgHHHSgxqrZGXYb2-uCrCtCc) | Pneumonia | Pneumonia | Fever;  Cough | Piperacllin/tazobactam;  Lineizoid | Pleural fluid: Acinetobacter *baumannii*  Sputum: Acinetobacter *baumannii* | None | Multiple Chrs Del/Dup |

Chr, chromsome; Dup, duplication; Del, deletion
